# The Current and Future Role of Technology in Respiratory Care

**DOI:** 10.1007/s41030-022-00191-y

**Published:** 2022-04-26

**Authors:** Persijn Honkoop, Omar Usmani, Matteo Bonini

**Affiliations:** 1 Dept of Biomedical Data Sciences, Section of Medical Decision Making, Leiden University Medical Centre, Leiden, The Netherlands; 2 National Heart and Lung Institute (NHLI), Imperial College London, Guy Scadding Building, Dovehouse Street, London, SW3 6LY UK; 3 Department of Cardiovascular and Thoracic Sciences, Università Cattolica del Sacro Cuore, Rome, Italy; 4 Department of Clinical and Surgical Sciences, Fondazione Policlinico Universitario A. Gemelli–IRCCS, Rome, Italy

**Keywords:** Digital, Technology, Telemedicine, Smart-inhaler, Artificial intelligence

## Abstract

Over the past few decades, technology and improvements in artificial intelligence have dramatically changed major sectors of our day-to-day lives, including the field of healthcare. E-health includes a wide range of subdomains, such as wearables, smart-inhalers, portable electronic spirometers, digital stethoscopes, and clinical decision support systems. E-health has been consistently shown to enhance the quality of care, improve adherence to therapy, and allow early detection of worsening in chronic pulmonary diseases. The present review addresses the current and potential future role of major e-health tools and approaches in respiratory medicine, with the aim of providing readers with trustful and updated evidence to increase their awareness of the topic, and to allow them to optimally benefit from the latest innovation technology. Collected literature evidence shows that the potential of technology tools in respiratory medicine mainly relies on three fundamental interactions: between clinicians, between clinician and patient, and between patient and health technology. However, it would be desirable to establish widely agreed and adopted standards for conducting trials and reporting results in this area, as well as to take into proper consideration potentially relevant pitfalls related to privacy protection and compliance with regulatory procedures.

## Key Summary Points

**Table:** 

Innovation technology and artificial intelligence are significantly changing the field of healthcare.
E-health is a term to describe a broad range of digital technologies and interventions used by a variety of stakeholders, across different settings.
Literature evidence shows that e-health improves the quality of care and the adherence to therapy, as well as allows early detection of worsening in chronic pulmonary diseases.
Widely agreed and standardized endpoints for conducting trials and reporting results on the role of technology in respiratory medicine are needed.
Potential pitfalls related to e-health interventions, such as patient privacy protection, data fishing, and non-compliance with regulatory positions, should be carefully considered.

## Introduction

Over the past few decades, technology and improvements in artificial intelligence (AI) have dramatically changed major sectors of our day-to-day lives, including the field of healthcare.

Thanks to the spread and easy availability of several innovative technological devices and systems, it is in fact at present possible to obtain, collect, and analyze vast amounts of data from individual patients, leading to improved and more targeted healthcare solutions. Such an approach of “Precision Medicine” has allowed public health policies and decision-making processes to adopt a delivery model that focuses on tailored traits, improving disease diagnosis and management, as well as helping to reduce the relevant burden of chronic respiratory diseases.

**Fig. 1:**
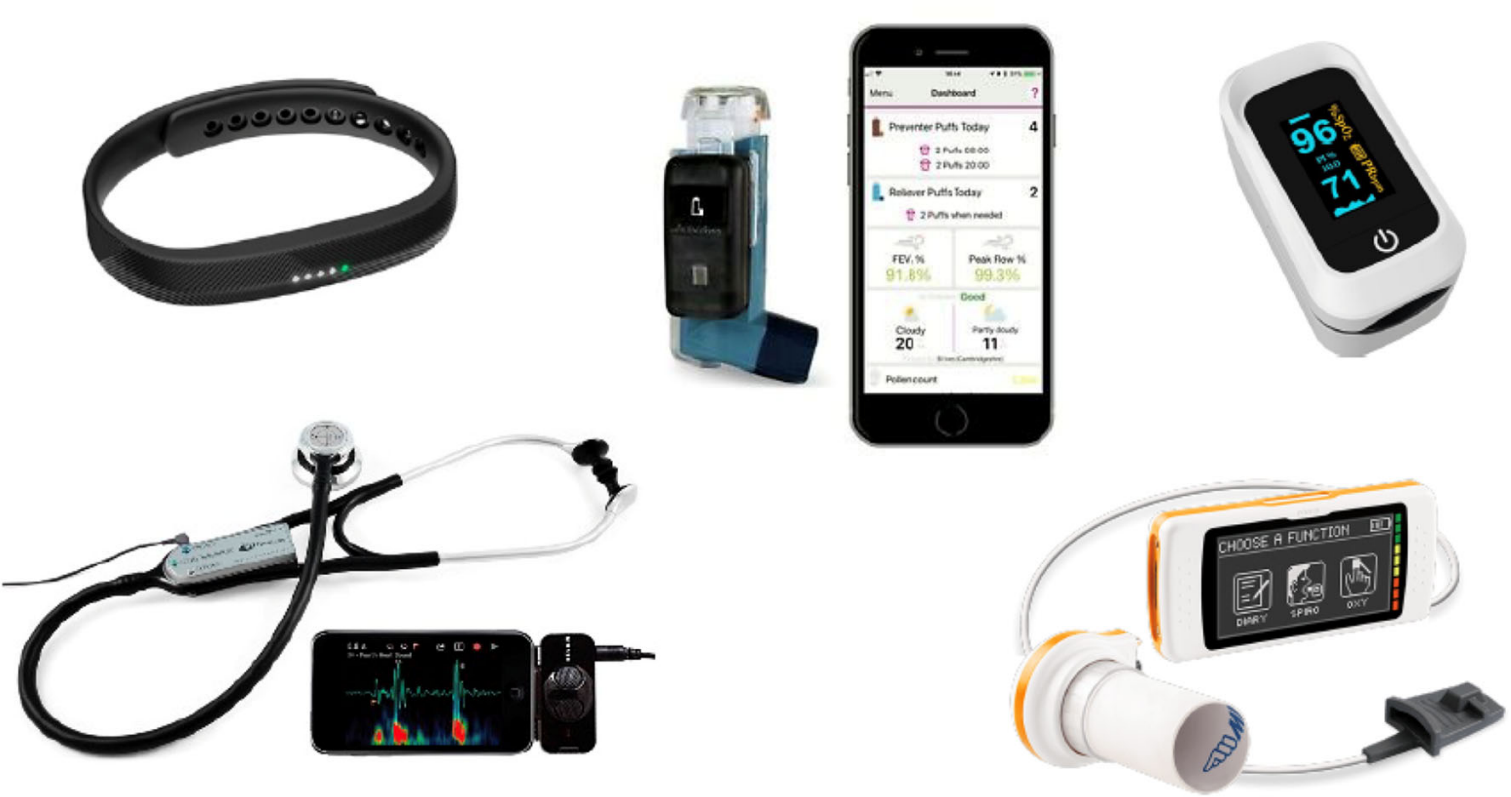
E-health tools

Electronic health (e-health) is a term coined in the late 1990s, to describe a broad range of digital technologies and interventions used by a variety of stakeholders, across different settings [1]. E-health includes a wide range of subdomains, such as mobile health, telemedicine, medication trackers, wearables, digital stethoscopes, and clinical decision-support systems (Fig. 1). E-health has been consistently shown to enhance the quality of care, improve adherence to therapy, and allow early detection of worsening in chronic pulmonary diseases [2]. In respiratory care, e-health has also been reported to empower patients with tools and skills to become optimal self-managers. Indeed, e-health allows proactive care by providing easy to use and personalized action plans. Furthermore, healthcare professionals may find e-health interventions valuable channels to actively interact with patients and to facilitate information exchange. In addition, the use of e-health is becoming widespread in the pharmaceutical industry and in research settings. Indeed, rather topically, with the public health emergency and the need to limit in-person appointments while maintaining access to care due to the COVID-19 pandemic, e-health services have significantly increased, giving the potential to provide patients with improved awareness of their pathological condition and foster their involvement in the care pathway. E-health programs are widely used in respiratory diseases, [3, 4] particularly in patients affected by chronic conditions, such as chronic obstructive pulmonary diseases (COPD), asthma, and interstitial lung diseases [5]. Remote patient monitoring involves continuous evaluation of patients’ clinical status, through real-time recordings or via analysis of functional parameters and vital signs remotely collected. In this framework, tools, such as wearables, smart inhalers, and digital stethoscopes, are often connected continuously with mobile applications in order to deliver medical expertise depending on patients’ needs.

The present review addresses the current and potential future role of major e-health tools and approaches in respiratory medicine, with the aim of providing readers with reliable and cutting-edge evidence to increase their awareness on the topic, and to allow them to optimally benefit from the latest innovation technology.

This article is based on previously conducted studies and does not contain any new studies with human participants or animals performed by any of the authors.

## Role of Technology in Respiratory Medicine

### Wearables

The concepts of “ubiquitous” and “pervasive” human wellbeing monitoring are becoming a reality as a consequence of the important advances in sensors, miniaturized processors, body area networks, and wireless data transmission technologies, allowing the assessment of physical, physiological, and biochemical parameters in different environments without restriction of activity [6]. “Wearable” means whatever a subject can wear, such as watches, patches, or devices, without limiting daily activities or restricting mobility. Presently, there are an overwhelming number of fashionable wearable devices, but not all of them are capable of reliably measuring the health status of the wearer [7]. Conversely, there are plenty of sensors that measure physiological parameters, but are not in a wearable form. Wearable biomedical sensors are therefore a subset of devices that are able to both measure physiological parameters and be worn. Biomedical wearables are commonly worn on the wrist, earlobe, finger, or trouser belt. Several products have been tested for assessing and monitoring both individual (i.e., respiratory rate, oxygen saturation, heart rate, glycemia, physical activity) and environmental parameters (i.e., air temperature and humidity, allergen and pollutant air concentration) [8].

Pulse oximeters, for example, measure light absorption [which depends on the levels of oxygenated (oxyhemoglobin) and deoxygenated blood] through an electronic processor and a pair of small light-emitting diodes illuminating the skin [9]. Pulse oximetry is commonly measured on the spot, but can also be continuously checked during daily activities, and at night to reveal potential effort-driven or nocturnal desaturations. Pulse oximetry has a crucial value in the assessment and management of acute dyspnoea, both in primary and secondary care. Currently, however, there is little evidence to support or refute the use of home pulse oximetry in the self-management of asthma, although the oxygen saturation (SpO_2_) value is considered a valuable marker to identify patients with asthma exacerbations at risk of respiratory failure, and who need to be promptly hospitalized [10]. Similarly, in COPD patients, low SpO_2_ has been shown to be a reliable marker of symptom worsening and the occurrence of an exacerbation episode [11, 12]. Interestingly, more recent bio-wearables tested on COPD patients with SpO_2_ measured continuously have generated personalized alerts, based on intelligent algorithms, that distinguish physiological oxygen fluctuations from real signs of disease decline [13]. Home telemonitoring systems, including pulse oximetry measurements, have also been tested with benefits in patients affected by interstitial lung diseases, and in particular in idiopathic pulmonary fibrosis (IPF) [14]. Of current relevance, home pulse oximetry has become an essential monitoring tool during the COVID-19 pandemic, helping to assist self-isolated patients, and to detect early deterioration of the pulmonary function [15].

In addition to blood oxygenation, pulmonary ventilation provides key information on abnormal respiratory patterns, especially in patients at risk of sudden deterioration in their clinical condition [16–18]. Respiratory rate is considered one of the most reproducible indirect parameters of pulmonary ventilation, and can be measured by body surface wearable sensors placed on chest bands, shirts, bras, or trouser belts [19–21].

Moreover, wearable devices have garnered significant attention as a safe and cost-effective healthcare device for tracking physical activity; this being highly correlated with health outcomes, such as disease morbidity, hospitalization and mortality [22]. Given that most respiratory diseases are associated with cardiovascular comorbidities and muscular deficits, wearable devices have become a valuable tool to promote an active lifestyle. Exercise tolerance can be measured through several endpoints, such as distance walked/run, diaries and questionnaires, metabolic activity, heart rate, and body temperature. Remarkably, “fitness trackers” (i.e., Fitbit) can suggest personalized rehabilitation or training programs depending on the user’s goals and needs [23–26].

Several wearable devices are being developed to monitor indoor and outdoor air quality, as well as other environmental data. Data can be transmitted via Bluetooth or wi-fi to mobile phones to create maps of air quality. This could be particularly helpful, for example, in atopic asthmatic patients, who should avoid irritants, allergens, and pollutants to minimize the risk of developing exacerbations [27] There are several barriers to the implementation of wearables in daily clinical practice. We have already mentioned the reliability of the results [7]. There is also an issue with interoperability, how to get the data of these devices into the electronic medical record (EMR) that healthcare professionals (HCPs) use, and, furthermore, how to ensure the HCPs are not flooded with data. A well=designed implementation strategy is needed before introducing these to regular medical care [28, 29].

### Smart Inhalers

Inhaled medication is the keystone in the management of patients affected by chronic pulmonary diseases [30]. Electronic monitoring of adherence to inhaler therapy has been feasible since about the 1980s, when the 'Nebuliser Chronolog' was first adopted in the academic setting [31]. However, it is only in the past 10 years that the electronics have become sufficiently portable, reliable, and affordable to allow their extensive diffusion in the market. Such progress has been paralleled by significant improvements in wireless technologies and mobile computing, allowing information to be swiftly transferred from the inhaler to a personal device, following remote analysis, and shown to the user in a clear and attractive way.

It is well known that unsatisfactory adherence and poor inhalation technique to prescribed pharmacological treatment is one of the most relevant causes of lack of disease control among patients affected by chronic respiratory diseases. This has been repeatedly associated with negative clinical outcomes, impaired quality of life, and increased healthcare costs [32, 33].

Electronic monitoring devices, also called “smart inhalers”, seem to have a crucial role in objectively monitoring adherence, and consequently providing precious real-time feedback to patients. Smart inhalers take advantage of a sensor attached to or integrated in the inhaler, and designed to connect, mostly via a Bluetooth system, to a mobile application. These devices can offer several functions, from simple tracking tools, only counting the usage of an inhaler, to more sophisticated devices that provide personalized alerts. Indeed, smart inhalers can remind patients to use their inhalers and provide personalized feedback depending on the time of use, inhalation technique, correlation with peak flow values, or questionnaire scores. Such information can be used, not only to detect and correct patient habits but also to guide physician decision processes in adjusting management. Several trials have been conducted testing smart inhaler use among COPD and asthma patients, showing adherence improvement and concomitant overall decrease in exacerbation rate and improved lung function [32, 34, 35].

In 2007, Charles and co-authors [36] published the results of a randomized trial performed in more than 100 asthmatic patients, aiming to assess the impact of an audio-visual reminder feedback (AVRF). All the participants were given a smart inhaler device, although, in the control arm, this was exclusively adopted to record the compliance to treatment as the primary outcome. Study findings revealed a significantly improved adherence to the prescribed therapy in the intervention group over the observational period (88% vs. 66% of total intended doses).

The relevance of HCPs providing information concordant to those captured by smart inhalers was furthermore assessed in a randomized pilot trial, enrolling children referred to a respiratory outpatient clinic. Study results showed that adherence rate was significantly increased in children receiving feedback in comparison to those that did not, over a 4-month period (79% vs. 58%) [37].

In addition, the Study of Asthma Adherence Reminders, combining the potential of AVRF to that of personalized support offered by physicians to patients, strengthened the evidence of a marked difference in compliance between subjects assigned to the investigational arm and those allocated to the control group, although the study power was not determined in advance, this being just a secondary endpoint [38].

Interestingly, Moran and co-workers, with the aim of evaluating the relationship between standard measures of adherence and more recently suggested scores/parameters, carried out a longitudinal observational study in a large sample of subjects diagnosed with COPD. Study results revealed that, despite 59 59% concordance between long-acting beta agonists used in combination with inhaled corticosteroid prescriptions and actual use, as measured by the number of delivered doses, a lower number of inhalations (47%) had actually been administered within the proper time-window [39].

Advances in smart inhaler technology also permitted feedback on inhalation techniques. Such an innovative way of monitoring has importantly minimized the risk of critical errors when using both pressurized metered-dose inhalers and dry-powder inhalers, also allowing a more proper calculation of a reliable degree of adherence. In regard to this, smart inhaler feedback has been reported to be superior even to intensive education when referring to the share of doses taken appropriately and timely, thanks to a lower risk of over- or missed dosing, as well as of mistakes in the inhalation technique [40]. Similar to wearables, smart inhalers also face barriers in implementation. For smart inhalers, specifically, the additional costs of the device can be an important barrier. Also, monitoring of adherence can be perceived as threatening by a patient, so it needs a proper introduction and an approach of positive feedback.

### Portable Electronic Spirometers

Pulmonary function tests are useful references to optimal diagnosis and monitoring of patients with chronic respiratory diseases. Particularly for asthmatic and COPD patients, spirometry represents the gold standard test for disease diagnosis and classification, as well as a valuable aid for assessing disease severity, prognosis, and management [41]. Despite its crucial role, spirometry is not performed as much as it should be, due to several reasons, including bulky devices, complex interpretation software, need for frequent calibration, maintenance, and supply costs, and special training for performing and interpreting tests. With the increasing demand for high-quality healthcare services, the advent of innovative and portable medical devices that can be used remotely and in resourceless conditions is improving delivery and quality of care. Multiple randomized controlled trials and systematic reviews have shown efficacy in improving asthma control and quality of life, while reducing the rate of severe asthma exacerbations [42, 43].

Following an earlier report of self-monitoring of pulmonary function using a home spirometer, [44] a study investigated the reliability, feasibility, and impact of home-based measurement of forced vital capacity (FVC) and dyspnea in a US population of IPF patients [45]. Another study conducted in a specialized center in the Netherlands confirmed the feasibility of a home monitoring program including real-time wireless home spirometry in IPF patients [46].

Carpenter and coworkers recently conducted an elegant systematic review of commercially available portable electronic spirometers designed for asthma patient use [47]. The search strategy yielded 36 devices, 16 of which met inclusion criteria. Although all the devices were designed for use by patients with asthma, most were also suitable for other respiratory diseases. For instance, ten devices (62.5%) targeted COPD patients, six (37.5%) cystic fibrosis, and five (31.3%) bronchitis and emphysema. Three devices (18.8%) were also marketed to monitor lung function after lung transplant surgery. All the 16 portable spirometers provided testing for peak expiratory flow (PEF) and forced exhaled volume in the first second (FEV1), while 13 (81.3%) also allowed patients to measure FVC. Interestingly, half of the devices measured FEF25–75, which is an indirect endpoint of the extent to which the smaller airways are affected [48, 49]. Ten devices provided graphical representations of lung function results. Seven gave patients immediate visual or audio feedback on whether they had performed the test correctly. Six devices had a traffic light system indicating whether patients’ pulmonary status was in the red (danger zone), yellow (caution), or green (safe) zone. Almost all the spirometers, either directly or via their associated apps, allowed patients to share lung function test results with their healthcare provider. Of note, however, only four devices had obtained approval from the FDA and the majority of them (65%) did not provide any information regarding how data security was addressed, which is a major barrier to implementation. It is also uncertain whether the other devices will eventually submit an application for FDA approval, since it is an arduous process. Moreover, it has to be repeated for every (major) change to the device. This is problematic for companies working according to a continuous improvement principle, whereby they frequently tweak the devices. Although it is understandable that very frequent resubmissions for FDA approval are bypassed from a company perspective, it limits their applicability, and these devices cannot be recommended by clinicians. Similar to wearables and smart inhalers, a proper integration of the data these devices generate into the EMR will be essential. For implementation to be a success, these data should aid a clinician in decision-making, rather than be overwhelming [50, 51].

A commonly used device in clinical practice is the PIKO-1 (Pulmonary Data Services; Ferraris Medical, Hertford, UK), which is able to measure both PEF and FEV1. The device has a built-in memory which stores the last measurements performed, and reports a comparison of each measurement to the patient’s reference value for that parameter. Data are also automatically transferred to a cloud platform which provides real-time feedback to the patient through a specifically designed app. It has been shown that, when regularly adopted, PIKO-1 and other similar devices can facilitate the patient's awareness of the disease, and encourage cooperation with the GP or respiratory physician [52].

### Digital Stethoscope

The stethoscope was invented in 1818 by a French physician, René Laennec [53]. Lung sounds acquired by stethoscopes are extensively used in diagnosing and differentiating respiratory diseases. However, despite the huge effort made over recent decades to interpret these sounds and to identify diseases associated with certain patterns, effective stethoscope use is limited to the individual experience of practitioners. Electronic stethoscopes represent an innovative method to increase the accuracy of lung auscultation, by amplifying and recording lung sounds and transforming them into digital signals that can be later processed and shared. The digital stethoscope consists of three different modules, data acquisition, pre-processing, and signal processing, before the listener can appreciate the auscultated sound. The data acquisition module involves a microphone and a piezoelectric sensor. It is responsible for filtering, buffering, and amplification of the auscultated sounds, as well as the conversion of the acoustic sound to a digital signal. The pre-processing module filters the digital signal and removes any artefacts. These digital data are then forwarded to the signal-processing module, which packages the information in a higher-order classification, and clusters the data for a clinical diagnostic decision.

Gurung et al. performed a meta-analysis of studies aiming to understand the prognostic power of combining digital pulmonary auscultation with computer-based algorithms, showing that the specificity and sensitivity of identifying abnormal pulmonary sounds, using computer- based algorithms, were 85% and 80%, respectively [54].

In an ongoing, multi-center clinical trial (clinicaltrials.gov identifier: NCT03503188), digital lung sounds and basic patient characteristics are being prospectively collected from patients with IPF and from symptom-matched control subjects. A well-established teaching device for lung sounds will be used as a reference [55].

Further studies have tried to create a unique and standardized protocol for the correct use of digital stethoscopes, starting from the identification of specific chest auscultation points and the classification of sounds based on score grading scales. Nevertheless, there is the need of further and deeper analysis among a wider cohort of patients that could lead to record and classify a greater range of pathological sounds. This is an essential condition for the creation of a complete sound database that can enhance and improve the sensibility and specificity of electronic auscultation process [56, 57]. Until that is created, the limited reliability is a major barrier to implementation. Also, users will have to get used to the sounds created in this way, because they will be (subtly) different to what doctors are used to. A lot of thought will also need to go into the proper implementation into the organization of care. Of course, these sort of devices might allow for long-distance consultations, which would provide an excellent solution for more rural areas. However, care still needs to be organized properly, and the doctor responsible will need to have sufficient data available to make an informed clinical decision [58].

### Artificial Intelligence (AI) Diagnosis

Recent years have seen an explosion of interest in the use of artificial intelligence and machine-learning (ML) techniques in medicine [59]. AI may be defined as “the theory and development of computer systems able to perform tasks normally requiring human intelligence, such as visual perception, speech recognition, decision-making, and translation between languages”; ML is a subfield of AI in which statistical models are used to learn patterns from data to accomplish a specific task. Such a phenomenon has been driven by the development of deep neural networks (DNNs) that can process complex input data and output a classification, such as ’normal’ or ’abnormal’. DNNs are ’trained’ to use large banks of data that have been assigned the correct labels. DNNs have shown the potential to equal or even surpass the accuracy of human experts in pattern recognition tasks, such as interpreting medical images or biosignals [60–62] Within respiratory medicine, the main applications of AI and machine learning thus far have been the interpretation of thoracic imaging, lung pathology slides,and physiological data, such as pulmonary function tests [63, 64].

ML has been used for instance to create an algorithm for the classification of fibrotic lung diseases using high-resolution computed tomography (HRCT) of the lungs to distinguish between usual idiopathic pneumonia (UIP) and non-UIP patterns, with comparable accuracy to an expert radiologist. In particular, CALIPER (Computer-Aided Lung Informatics for Pathology Evaluation and Rating) analyzes and evaluates the severity of parenchymal pulmonary abnormalities (honeycombing, reticular abnormalities, ground-glass opacities, emphysema) and progression over time in HRCT [65, 66].

**Fig. 2:**
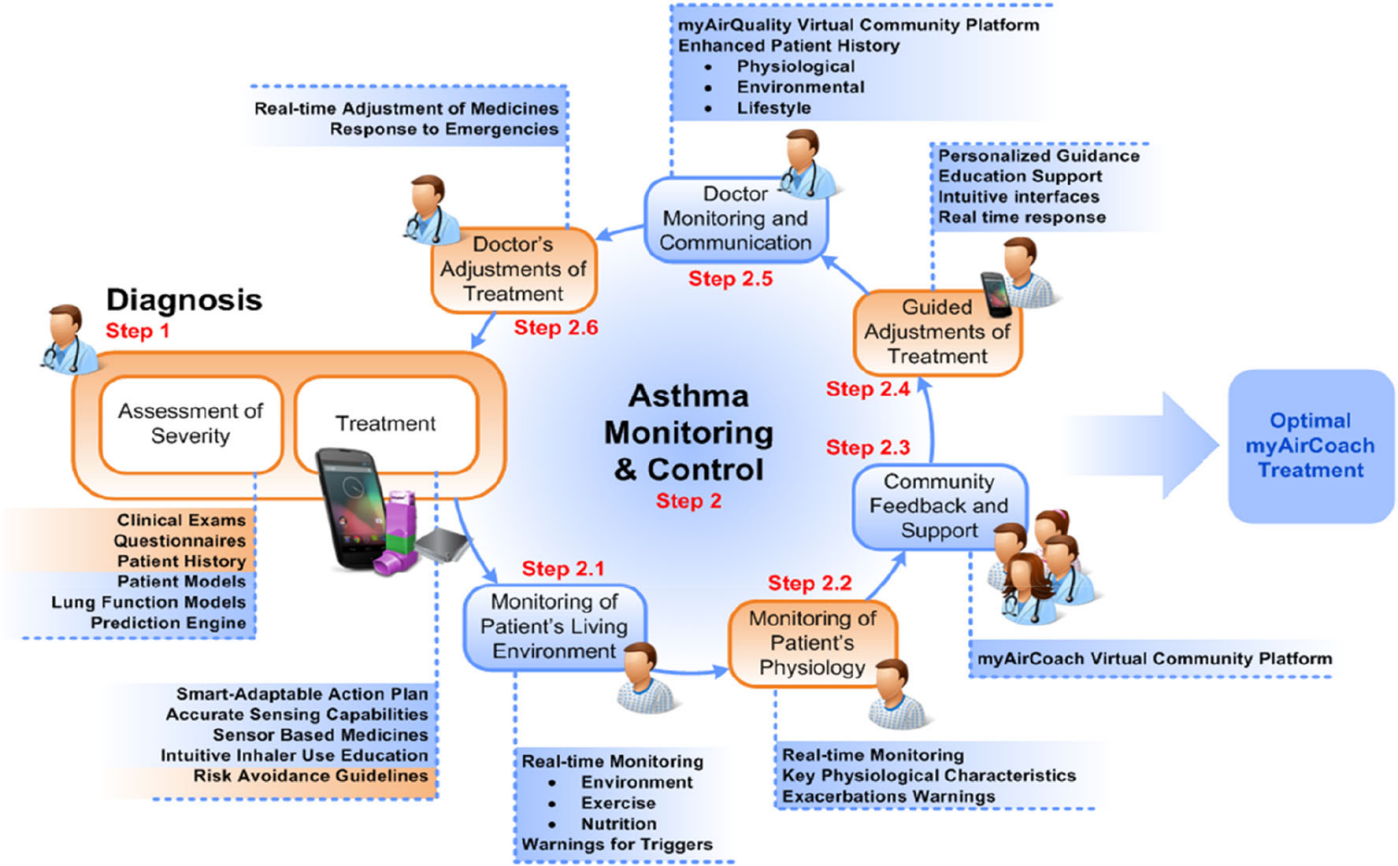
myAirCoach management approach

Furthermore, an integrated home-monitoring system (myAirCoach) has recently been tested with the aim of assessing its clinical effectiveness and acceptance, on top of usual care, in patients with asthma using inhalation medication [67]. The myAirCoach system consists of an inhaler adapter, an indoor air-quality monitor, a physical activity tracker, a portable spirometer, a fraction exhaled nitric oxide device, and an app (Fig. 2). The primary outcome has been asthma control, while secondary outcomes are exacerbations, quality of life, and patient acceptance. Subjects were recruited in 2 separate studies: in the first, 30 participants were randomized to either usual care or myAirCoach support for 3–6 months, while in the second study, 12 participants were provided with the myAirCoach system in a 3-month before–after study. Using the myAirCoach support system significantly improved asthma control and quality of life, with a reduction in severe asthma exacerbations. Participants reported positive attitudes toward the system [68]. AI presents us with several new barriers. There are concerns regarding privacy and security of data, and questions regarding liability. AI can also impact negatively on patient autonomy [69].

## Conclusions

Given the scarcity of resources for managing chronic lung diseases, it is particularly important to consider the potential added value of novel digitization concepts in respiratory medicine. However, before implementation, potential interventions need to be assessed for their efficacy and cost-effectiveness, and with more bonding between sectors and disciplines. Technology interventions have been proven to be acceptable to respiratory patients and physicians, and the relative ubiquity of mobile Internet- and Bluetooth-connected devices makes real-time monitoring of pulmonary diseases extremely feasible in everyday life. Overall, the potential of technology tools in respiratory medicine relies on three fundamental aspects: the interaction between clinicians, the interaction between clinician and patient, and the interaction between patient and the health technology. Moreover, personalized technological approaches, as well as innovative care pathways to patients, could significantly reduce healthcare costs. However, an effective digital intervention must fulfil several important design criteria. Both inhaler devices and software should be intuitive and easy to use, and hardware should be unobtrusive, with accurate and objective measurement of adherence and other parameters. Motivating patients to take an active role in managing their condition, and maintaining their interest in doing so, is of paramount importance in designing a successful future digital intervention. Lastly, the current lack of proper study power assessments and the challenges in establishing a minimal clinical difference particularly for the patient-reported outcomes makes it hard to compare and fully interpret study findings. There is, therefore, the need to establish widely agreed and adopted standards for conducting trials and reporting results on the role of technology in respiratory medicine. These should also take into consideration potential pitfalls related to e-health interventions, such as patient privacy protection, data fishing, and non-compliance with evidence-based medicine, guideline recommendations, and regulatory board statements.
